# Comparison of a new MR rapid wash-out map with MR perfusion in brain tumors

**DOI:** 10.1186/s12885-024-12909-z

**Published:** 2024-09-12

**Authors:** Eya Khadhraoui, Leon Schmidt, Stefan Klebingat, Roland Schwab, Silvia Hernández-Durán, Georg Gihr, Harald Paukisch, Klaus-Peter Stein, Daniel Behme, Sebastian Johannes Müller

**Affiliations:** 1 Clinic for Neuroradiology, Otto-Von-Guericke-University Magdeburg, Leipziger Str. 44, D-39120 Magdeburg, Germany; 2 Department of Neurological Surgery, Göttingen University Hospital, Robert-Koch-Str. 40, D-37075 Göttingen, Germany; 3 Clinic for Neuroradiology, Katharinen-Hospital Stuttgart, Kriegsbergstr. 60, D-70174 Stuttgart, Germany; 4 Department of Neurosurgery, Otto-Von-Guericke-University Magdeburg, Leipziger Str. 44, D-39120 Magdeburg, Germany; 5 Stimulate Research Campus Magdeburg, Otto-Hahn-Str. 2, D-39106 Magdeburg, Germany

**Keywords:** Late enhancement, Contrast clearance, Wash out, MR perfusion

## Abstract

**Background:**

MR perfusion is a standard marker to distinguish progression and therapy-associated changes after surgery and radiochemotherapy for glioblastoma. TRAMs (Treatment Response Assessment Maps) were introduced, which are intended to facilitate the differentiation of vital tumor cells and radiation necrosis by means of late (20–90 min) contrast clearance and enhancement. The differences of MR perfusion and late-enhancement are not fully understood yet.

**Methods:**

We have implemented and established a fully automated creation of rapid wash-out (15–20 min interval) maps in our clinic. We included patients with glioblastoma, CNS lymphoma or brain metastases who underwent our MR protocol with MR perfusion and rapid wash-out between 01/01/2024 and 30/06/2024. Since both wash-out and hyperperfusion are intended to depict the active tumor area, this study involves a quantitative and qualitative comparison of both methods. For this purpose, we volumetrically measured rCBV (relative cerebral blood volume) maps and rapid wash-out maps separately (two raters). Additionally, we rated the agreement between both maps on a Likert scale (0–10).

**Results:**

Thirty-two patients were included in the study: 15 with glioblastoma, 7 with CNS lymphomas and 10 with brain metastasis. We calculated 36 rapid wash-out maps (9 initial diagnosis, 27 follow-up).

Visual agreement of MR perfusion with rapid wash-out by rating were found in 44 ± 40% for initial diagnosis, and 75 ± 31% for follow-up. We found a strong correlation (Pearson coefficient 0.92, *p* < 0.001) between the measured volumes of MR perfusion and rapid wash-out. The measured volumes of MR perfusion and rapid wash-out did not differ significantly. Small lesions were often not detected by MR perfusion. Nevertheless, the measured volumes showed no significant differences in this small cohort.

**Conclusions:**

Rapid wash-out calculation is a simple tool that provides new information and, when used in conjunction with MR perfusion, may increase diagnostic accuracy. The method shows promising results, particularly in the evaluation of small lesions.

## Introduction

The classic analysis of contrast dynamics in brain tumors in MRI is limited to contrast enhancement and contrast agent flooding using MR perfusion. MR perfusion is the current standard for the differentiation of tumor recurrence and treatment related changes in the follow-up of patients with high grade gliomas [1, 2]. It also plays a crucial role in the initial diagnosis, especially in distinguishing gliomas from CNS lymphoma [3].

The question of what happens to the contrast agent in the tumor and its surroundings at a later point in time has long been neglected in diagnosis finding. The basic concept of a late contrast clearance (> 60 min) for brain tumors was introduced by Zach et al. in 2012 [4].

It has been shown that this method called TRAMs (Treatment Response Assessment Maps) works very well in distinguishing tumor progression from radiation necrosis or pseudoprogression in glioblastoma [5], and helps to assess the response to therapy in CNS lymphomas [6] or metastasis [7]. A study from Satvat et al. [8] revealed that even a shorter waiting time (< 20 min) between the two T1-weighted sequences can produce meaningful results.

Firstly, we noticed a discrepancy between the protocols of both methods from Zach et al. and Satvat et al. and the current literature. In the initial protocols [4, 8], the first contrast-enhanced, T1-weighted sequence starts directly / 2 min after MR perfusion. But, the maximum Gadolinium enhancement of glioblastoma cells is described at approx. 3–8 min [9, 10] in rat models, and in metastasis even at approx. 10–15 min [11] after contrast agent applications.

For this reason, we initially incorporated a delay sequence into our MR protocol, as we wanted to ensure that the maximum enhancement of glioblastoma cells had already been achieved in the first T1-weighted sequence.

**Fig. 1:**
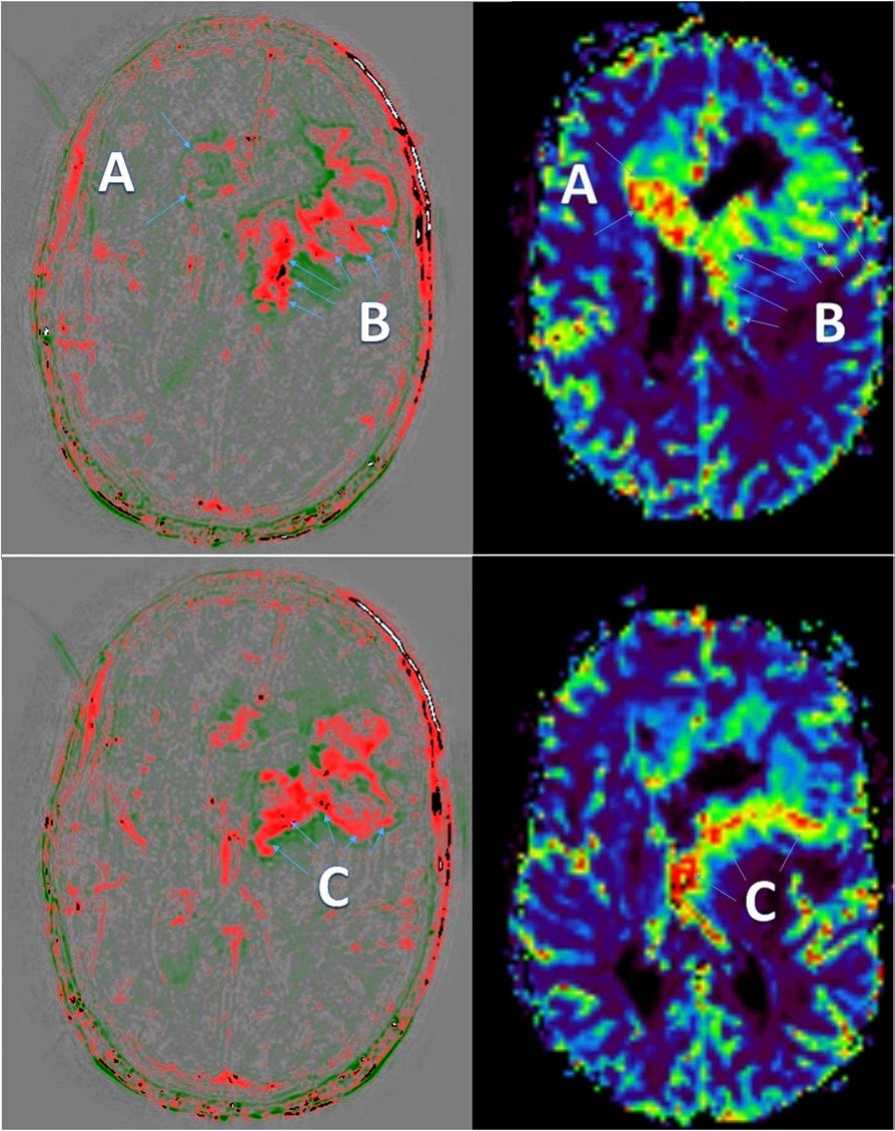
left: transverse rapid wash-out map, wash-out (red) and wash-in (green); right: transverse MR perfusion at the same level; two different heights of the tumor are cut above and below; **A** – mismatch between MR perfusion and rapid wash-out; **B** - partially matching tumor area; **C** – tumor area active in both MR perfusion and wash-out

Secondly, we optimized our calculated rapid wash-out maps for the semi-automated volumetric analysis with 3D slicer and normalized the maps for future processing using neural networks. We used a simple two-color-system with red for wash-out (active tumor cells / vessel) and green for wash-in (scar tissue / necrosis). Figure 1 demonstrates an example of the rapid wash-out maps and comparison with MR perfusion.

In this first part of our study, we wanted to demonstrate the feasibility and compare our rapid wash-out mapping with MR perfusion for glioblastoma, CNS lymphomas and brain metastases, both at initial diagnosis and while treatment.

## Methods

### Study design

This single-center observational study was ethically approved by the institutional review board. This study adhered to the 2013 Declaration of Helsinki. The institutional review board waived the requirement for informed consent because of the retrospective nature of the study and the analysis of anonymized patient data. All methods were performed in accordance with relevant guidelines and regulations.

### Participant population

We searched our PACS database for patients with valid rapid wash-out protocols between 01/01/2024 and 30/06/2024. We included only patients with a diagnosis of glioblastoma, CNS lymphoma or brain metastasis, and divided the found protocols into initial diagnosis and follow-up MRI.

### MRI protocol and technic details

We performed protocols on Siemens Sola 1.5T (Siemens Healthineers AG, Werner-von-Siemens-Str. 1, D-80333 Munich, Germany), Philips Achieva 3T and Philips Intera 1.5T (Koninklijke Philips NV, Amstelplein 2, Amsterdam, 1096 BC, Netherlands).

The detailed protocols are listed in Table 1. We acquired at three times the same T1-weighted sequence (native, 5 min and 25 min after contrast application): sagittal T1 MPRAGE on Siemens and transversal T1w 3D TFE on Philips MRI. If no contrast enhancement in the first contrast-enhanced T1-weighted sequence was detected, a neuroradiologist shortened the protocol accordingly to optimize clinical workflow.

**Table 1:** Detailed MR protocols

Sequence	Orientation	Time (sec) @ Philips Achieva 3T	Time (sec) @ Siemens Sola 1.5T	Time (sec) @ Philips Intera 1.5 T
3D T1 MPRAGE (Siemens)	sag		274	
3D TFE (Philips)	tra	258		280
→ contrast agent application				
MR T2* Perfusion	tra	67	151	85
T2 TSE	tra	218	147	213
3D T1 MPRAGE (Siemens)	sag		274	
3D TFE (Philips)	tra	258		280
3D T2 SPACE (Siemens)	tra		300	
3D T2 CISS (Philips)	tra	410		304
EPI DWI/ADC	tra	68	104	64
SWI	tra		334	
T2*	tra	210		175
3D FLAIR	sag	230	240	302
3D T1 MPRAGE (Siemens)	sag		274	
3D TFE (Philips)	tra	258		280
**Sums**				
Delay between contrast agent application and start of the first T1-weighted sequence		4.8 min	5.0 min	5.0 min
Delay between contrast enhanced T1-weighted sequences		15.3 min	16.3 min	14.1 min
Protocol length		**33.0 min**	**35.0 min**	**33.1 min**

*tra *transversal, *sag *sagittal, *TFE *Turbo field echo, *MPRAGE *Magnetization Prepared Rapid Acquisition with Gradient Echoes, *FLAIR *fluid attenuated inversion recovery, *EPI *Echo Planar Imaging, *DWI/ADC *Diffusion-weighted imaging / apparent diffusion coefficient, *CISS *Constructive Interference in Steady State, *SPACE *Sampling Perfection with Application optimized Contrasts using different flip angle Evolution, *SWI *Susceptibility weighted imaging

**Fig. 2:**
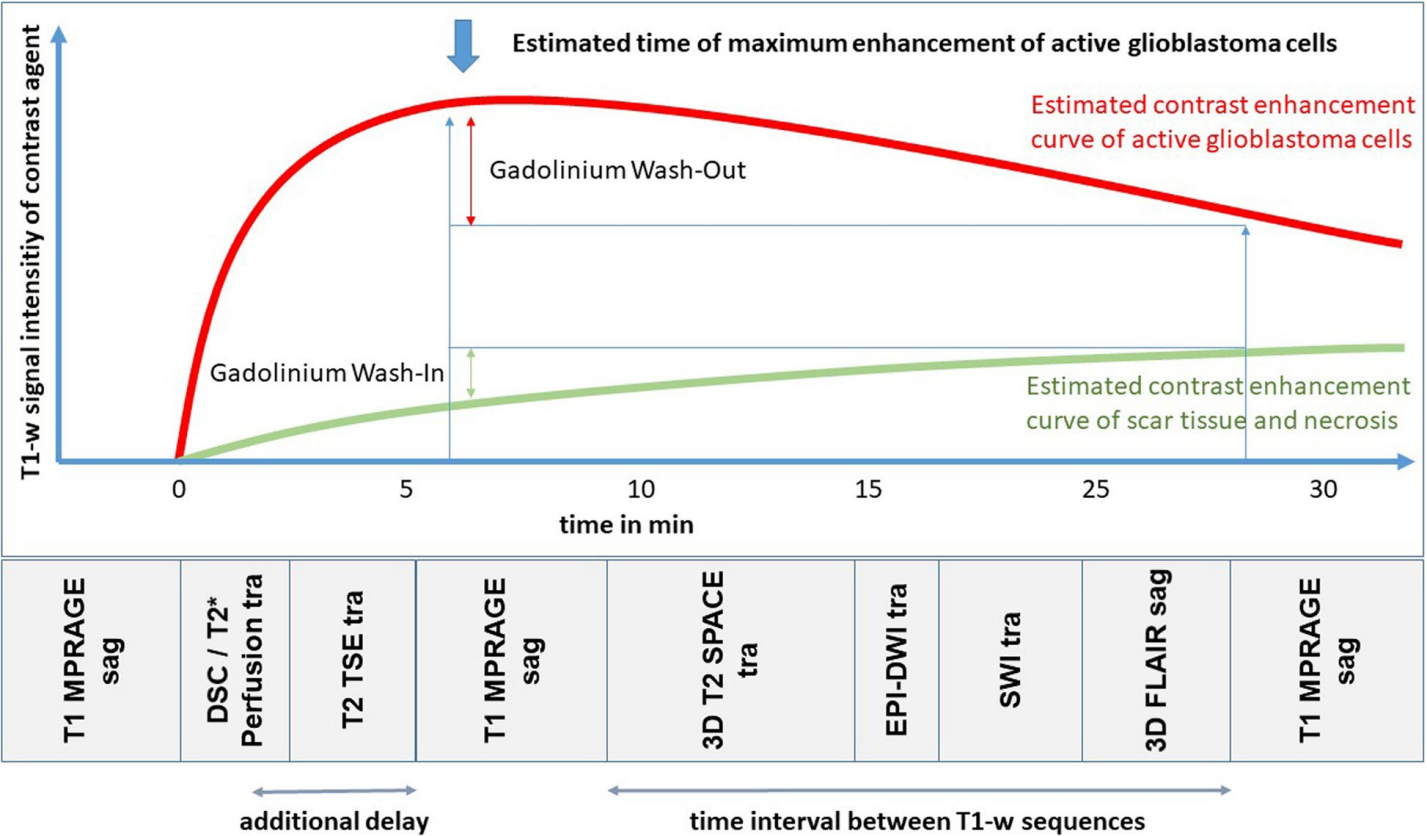
Time line of MRI protocol (bottom; exemplary for 1.5T Sola Siemens) and schematic overview of estimated contrast enhancement curves (top)

See Fig. 2 for a schematic overview of our protocol with estimated contrast enhancement curves.

Sequence parameters for T1-weighted imaging were TE = 3.02 ms, TR = 1680 ms, flip angle = 8° for sagittal T1 MPRAGE (slice-thickness 1 mm, resolution 1 × 1 mm, scan time 342 s, 1.5T Sola, Siemens); TE = 3.5 ms, TR = 7.9 ms, flip angle = 8° for T1w 3D TFE (slice-thickness 1 mm, resolution 1 × 1 mm, scan time 258 s, 3T Achieva, Philips); and TE = 5.9 ms, TR = 24 ms, flip angle = 25° for transversal T1w 3D TFE (slice-thickness 1 mm, resolution 1 × 1 mm, scan time 280 s, 1.5T Intera, Philips).

### Perfusion protocols and evaluation

Single dose MR perfusion was performed using manufacturer dependant DSC (Dynamic Susceptibility Contrast) T2* sequences (Siemens EP2D_Perfusion, Philips FFE_EPI_HR_Perfusion) without preload, and a bolus application of 0,1 ml Gadovist (Gadobust, Bayer Vital GmbH, Gebäude K56, D-51366 Leverkusen, Germany) per kg body weight with a flow of 3 ml / second. The perfusion curves were evaluated and rCBV was measured and calculated in the grey scale CBV maps as described by Cho et al. [12] with T2* MR Neuroperfusion Tool of Philips Intellispace or MR Neurology Perfusion Tool of Siemens syngo.via. We exported the rCBV maps to our PACS system for further analysis.

Volumetric measurements were performed for all tumor regions with contrast enhancement and elevated rCBV. Since rCBV-values of tumor regions can be low, especially in CNS lymphomas [13, 14], we choose a significant and optically distinguishable threshold of rCBV > 1.2. We did this to define as large a volume as possible using MR perfusion in this first feasibility study, although we knew that the optimal CBV threshold for the differentiation of pseudoprogression and recurrent high-grade gliomas seems to be between > 1.64 and > 1.75 [15]. We used the segment editor of the 3D slicer tool (www.slicer.org) to measure these volumes.

Sequence parameters for transversal T2* perfusion weighted imaging were TE = 31 ms, TR = 2840 ms, flip angle = 90° for EP2D_Perfusion (slice-thickness 4 mm, resolution 1.8 × 1.8 mm, scan time 151 s, 1.5T Sola, Siemens); TE = 40 ms, TR = 1552 ms, flip angle = 75° for FFE_EPI_HR_Perfusion (slice-thickness 4 mm, resolution 1.75 × 1.75 mm, scan time 67 s, 3T Achieva, Philips); and TE = 40 ms, TR = 1952 ms, flip angle = 75° for FFE_EPI_HR_Perfusion (slice-thickness 4 mm, resolution 2.33 × 2.33 mm, scan time 86 s, 1.5T Intera, Philips).

### Calculation of wash-out-maps

The wash-out masks are generated using Python 3.11 software developed in-house. First, both MRI series are converted to NIFTI format using the dicom2nifti library (version 2.4.10). The N4 bias field correction is applied to these images with a 3-fold reduction using SimpleITK (version 2.3.1). We then normalized both series to the same mean value and rigidly registered using the FLIRT class of the FSL library (version 6.0). Finally, the registered images are subtracted (series 1 minus series 2) and converted into both gray and RGB DICOM image series. For the RGB series, the used value range is first halved and then normalized in the middle to 127 out of 255. Negative subtraction values are colored green and positive values are colored red. Thus, higher values in the first MRI appear red while lower values appear green.

### Semi-automatically volumetric assessment

**Fig. 3:**
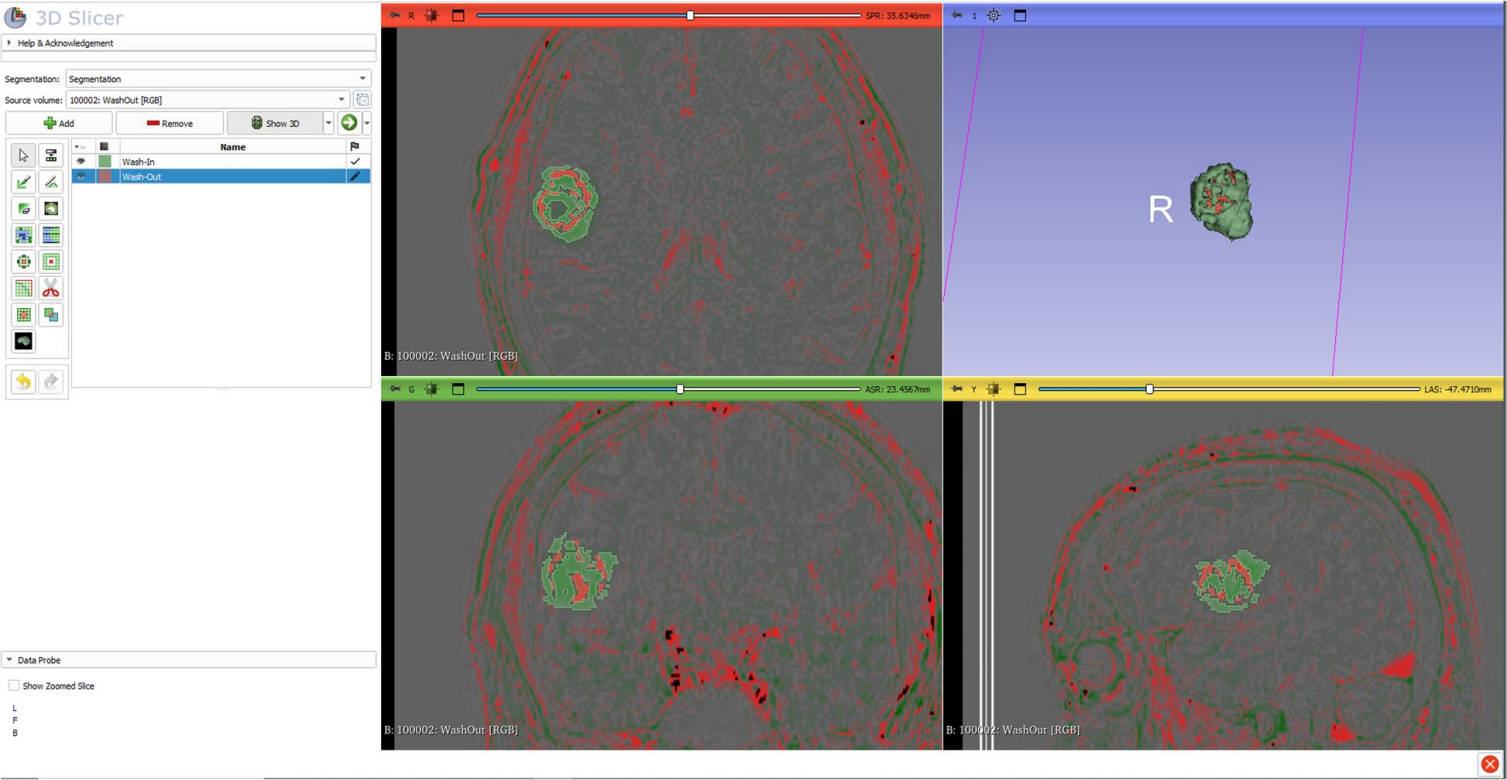
Screenshot of the 3D slicer with an example segmentation of a brain metastasis of a malignant melanoma three months after radiotherapy

We measured MR perfusion, wash-out and wash-in volumes for every patient via 3D slicer (level trace tool, slice by slice, semi-automatically). Figure 3 demonstrates an exemplary measurement of the volumes. The measurement were performed independently by to raters and finally averaged. ICC(2, k) was calculated. We also calculated the wash-out ratio as wash-out-volume / (wash-out-volume + wash-in-volume), previously described in a TRAMs study [16].

### Visual assessment and comparison

To avoid measuring unassociated volumes, the following additional ratings were used: We visually compared volumes segmented in MR perfusion and rapid wash-out maps slice per slice and rated the agreement of both on a 11-point-Likert scale 0–10 (0-100% agreement). Additionally, the following estimations were rated on a 11-point-Likert scale 0–10: (1) MR hyperperfusion not included in wash-out compared with whole hyperperfusion volume, and (2) wash-out not included in MR hyperperfusion compared with whole wash-out volume.

### Statistical analysis

We used Python3 (Version 3.11, matPlotLib) for statistical programming and histogram / image construction. We calculated the intraclass correlation coefficient (ICC, 2, k) using Python3 and the libraries “pandas” and “pingouin”. Interpretation of results was done following Koo and Li [17]. We used the Pearson coefficient for correlation analysis and Tukey’s Test for analysis of variance [18]. The significance level was set to 5%.

## Results

### Participants

**Fig. 4:**
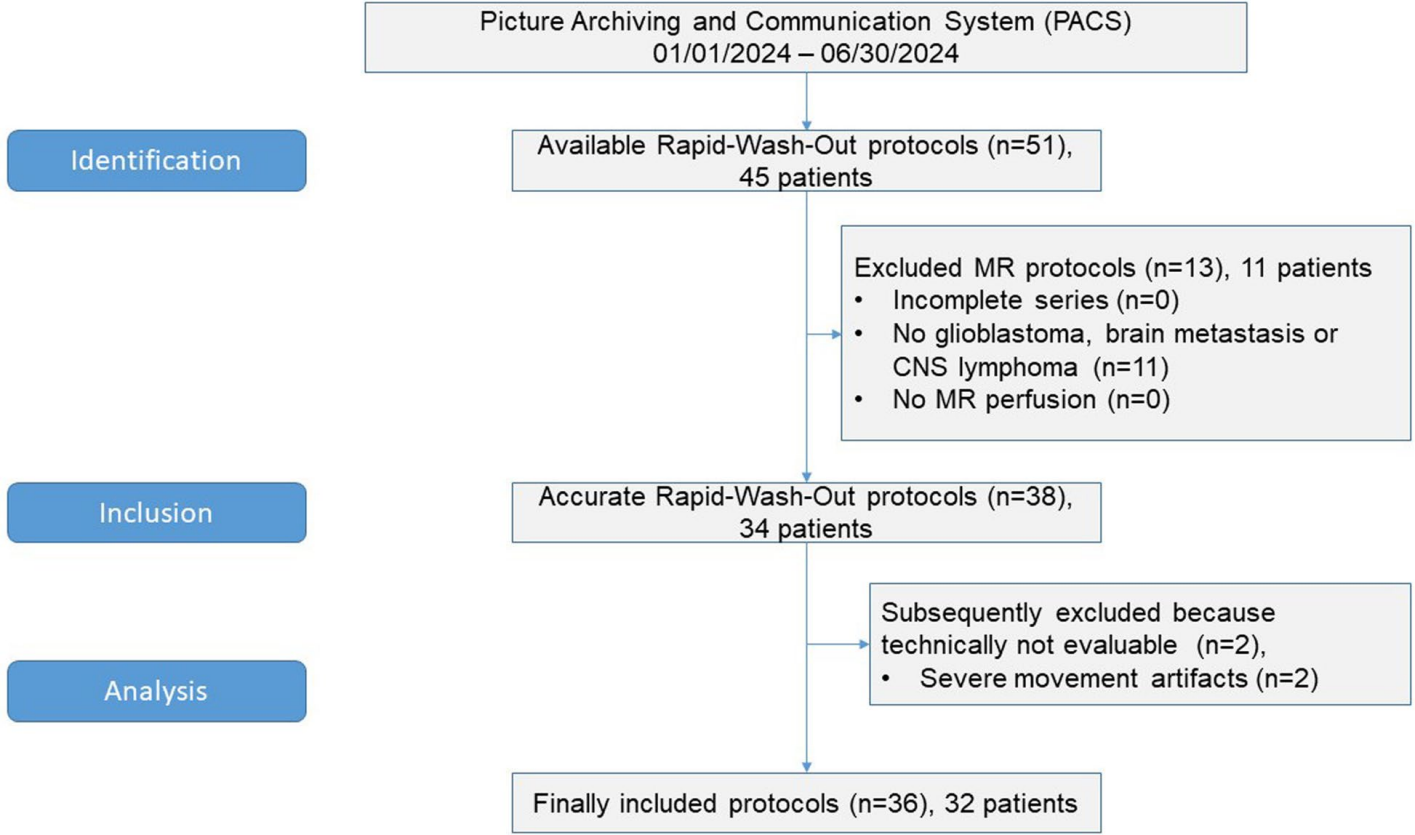
Inclusion flow chart

Our PACS search revealed 51 rapid wash-out protocols for 45 patients. We excluded 11 patients (13 protocols) without diagnosis of glioblastoma, CNS lymphoma or brain metastasis. At evaluation, we had to exclude two patients (2 protocols) because of severe movement artifacts. See Fig. 4 for a detailed flow chart. We formed six subgroups: initial diagnosis of (1) glioblastoma, (2) CNS lymphoma, (3) brain metastases; and follow-up MRI of (4) glioblastoma, (5) CNS lymphoma, and (6) brain metastases.

We finally included 32 patients (18 females, 14 males). Mean patients age was 67 ± 11 (mean ± standard deviation) years. Thirty-six rapid wash-out maps were included (11 on 1.5T Sola, 24 on 3.0T Achieva and one on 1.5T Intera).

### Calculation algorithm and time

The running time of the algorithms was mainly dependent on the registration task of the FLIRT class, which took between one and eight minutes. The remaining running time was less than one minute, so that all calculations took less than 10 min each. Thus, the calculation can be performed on a normal PC in almost real time.

### Histology

All included patients with high-grade glioma had a diagnosis of IDH-wildtype glioblastoma (WHO grade 4). The seven included CNS lymphoma patients suffered from a non-Hodgkin B-cell lymphoma. In the patients with initial diagnosis of brain metastases, we found the following histopathologies: adenocarcinoma of the lung, small cell lung cancer, transitional cell carcinoma and esophageal squamous-cell carcinoma. Pathologies for patient with brain metastases in follow-up scans were malignant melanoma, squamous cell carcinoma, small cell lung cancer and esophageal squamous-cell carcinoma.

### Visual comparison

**Fig. 5:**
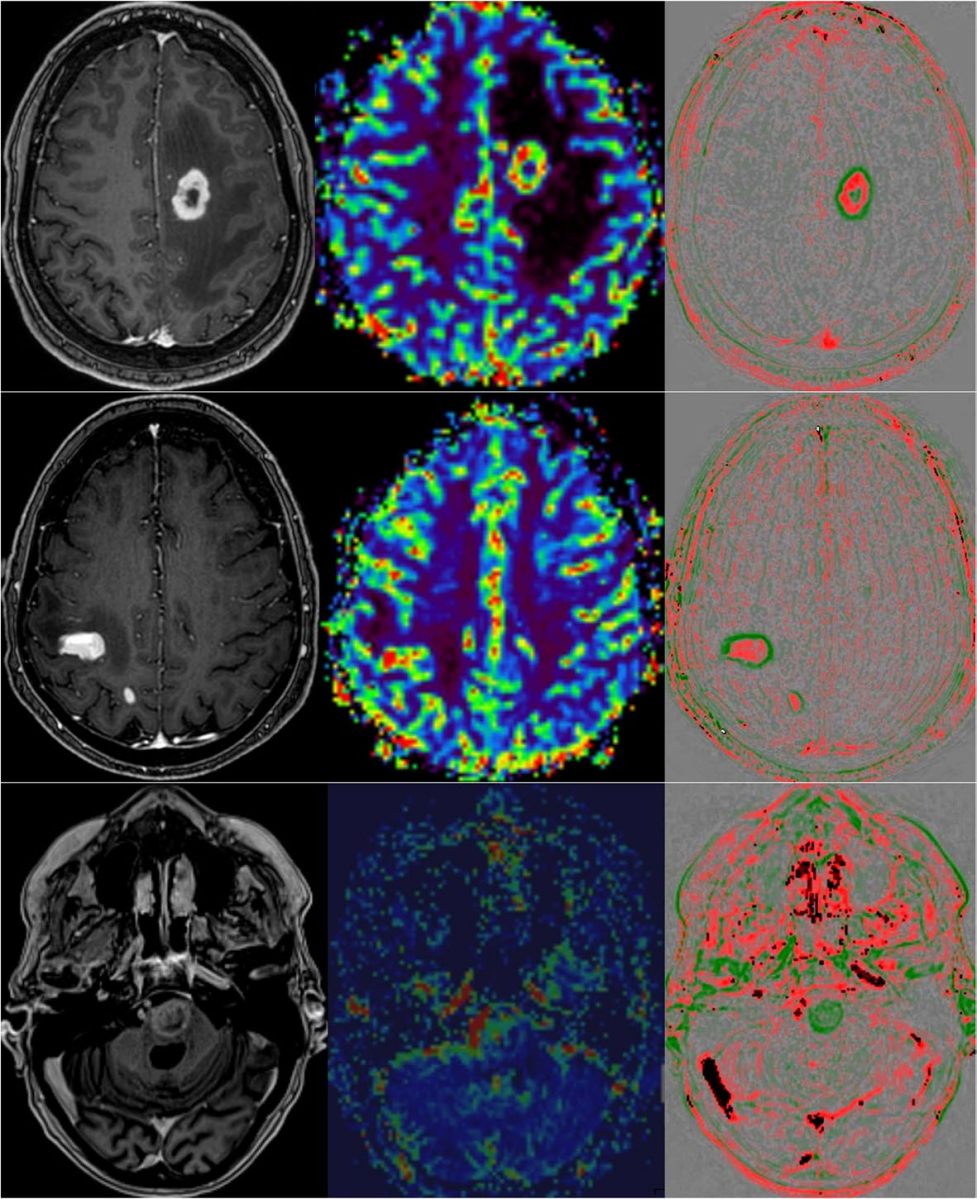
Examples of initial diagnosis (top: glioblastoma; middle: CNS lymphoma; bottom: brain metastasis of a small cell lung carcinoma). Left: contrast enhanced T1-weighted images; middle rCBV map of MR perfusion; right: rapid wash-out map

**Fig. 6:**
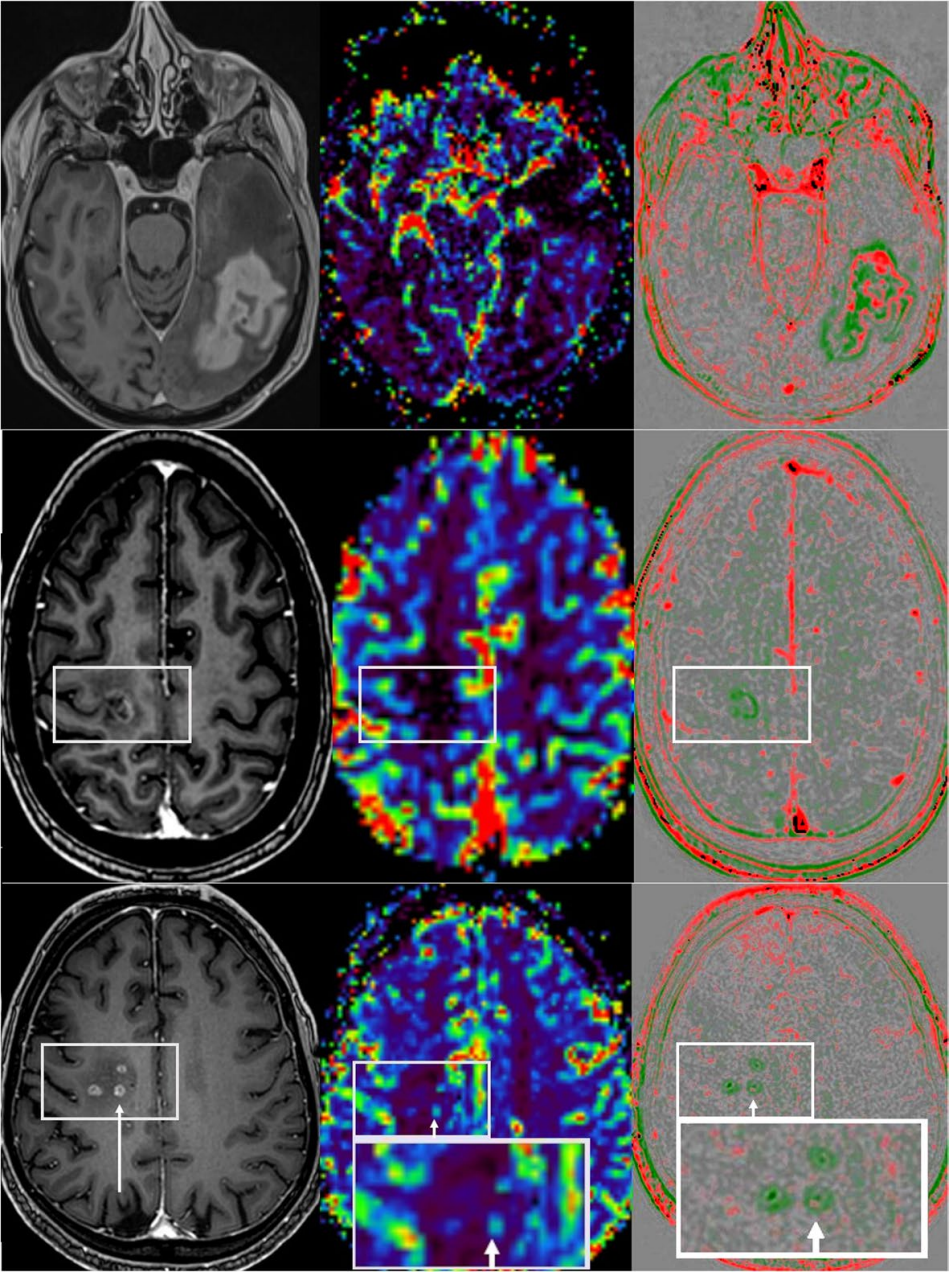
Glioblastoma therapy assessment. Top: 200% volume progress in 6-week follow-up. MR perfusion (middle) showed no local hyperperfusion. Rapid wash-out (right) revealed areas of increased rapid wash-out (red). Middle: Typical post-therapeutically changes without hyperperfusion and wash-out, but wash-in (green). Bottom: Radiation necrosis (3-month follow-up was stable) with less active tumor cells with bubble-like appearance of small contrast enhancing lesions and minimal hyperperfusion (arrow) as well as minimal ring-shaped wash-outs with red dot (arrow). Left: contrast enhanced T1-weighted images; middle rCBV map of MR perfusion; right: rapid wash-out map

We visually rated the agreement of MR perfusion and rapid wash-out per lesion. Table 2 demonstrates the results of this subjective evaluation. Since this rating was given per lesion and especially small lesions were often not detected by MR perfusion, large statistical differences were observed (especially for CNS lymphomas having small lesions in our cohort). While the MR perfusion was mostly almost completely contained in the wash-out regions, we also found large areas of wash-out that were not shown in the MR perfusion. Figure 5 reveals typical initial diagnosis imaging of DSC perfusion and rapid wash-out. During follow-up, the differences were not so notable, but this was also due to the fact that the CNS lymphomas in particular mostly showed a good response. Follow-up of glioblastoma are shown in Fig. 6.

**Table 2:** Mean ± standard deviation of the patient data and agreement of volumes on 11-point likert scale (0-100%)

	Patients *n*;	Lesions m	Sex (m: f)	Age in years	Visual agreement (in per ent) of rapid wash-out with MR perfusion	Visual MR perfusion not included in rapid wash-out in per cent	Visual rapid wash-out not included in MR perfusion in per cent
All patients	32	50	14:18	67 ± 11	64 ± 38	3 ± 11	48 ± 41
**Initial diagnosis**	9	16	5:4	67 ± 7	44 ± 40	8 ± 16	54 ± 42
Gb	4	4	2:2	63 ± 7	65 ± 44	5 ± 10	40 ± 43
CNS Lymphoma	1	6	0:1	76 ± 0	30 ± 40	0 ± 0	73 ± 34
BM	4	6	3:1	68 ± 5	45 ± 39	15 ± 22	47 ± 46
**Follow-up**	23	34	9:14	66 ± 12	75 ± 31	1 ± 5	44 ± 41
Gb	11	16	4:7	66 ± 14	73 ± 28	2 ± 8	51 ± 36
CNS Lymphoma	6	6	2:4	64 ± 8	80 ± 45	0 ± 0	20 ± 45
BM	6	12	3:3	63 ± 12	77 ± 32	0 ± 0	47 ± 46

*Gb *glioblastoma, *BM *brain metastasis

### Volumetric compare

Due to the different voxel sizes and the possible partial volume effect, the data should be viewed with caution. The results of volumetric measurement of MR perfusion and rapid wash-out maps are listed in Table 3.

**Table 3:** Mean ± standard deviation of the volumetric results

	Initial diagnosis			Follow-up		
subgroup	Gb	CNS Lymphoma	BM	Gb	CNS Lymphoma	BM
lesions	4	6	6	16	6	12
rrCBV * (mm³)	3.0 ± 2.9	2.0 ± 0.5	2.1 ± 3.4	2.0 ± 1.0	0 ± 0	1.2 ± 0.9
Perfusion volume (mm³)	2795 ± 2948	635 ± 718	354 ± 557	8164 ± 9400	0 ± 0	294 ± 544
Wash-Out volume (mm³)	2715 ± 2159	588 ± 741	360 ± 435	9499 ± 9270	33 ± 32	320 ± 480
Wash-In volume (mm³)	9117 ± 4755	2671 ± 3728	3172 ± 3531	20,746 ± 15,433	840 ± 751	2150 ± 2804
Wash-out ratio	25 ± 15%	14 ± 9%	13 ± 18%	23 ± 12%	2 ± 2%	10 ± 11%

*rrCBV *ratio of relative cerebral flood flow, *Gb *glioblastoma, *BM *brain metastasis; *-only measured if an area with contrast enhancement > 0.25 cm² was detected

We found a strong correlation between the measured volumes of MR perfusion and rapid wash-out maps with a Pearson correlation coefficient r of 0.92, (*p* < 0.001; R²=0.84).For wash-out ratios at initial diagnosis, the number of lesions was not sufficient to make a statistical statement. Wash-out ratios of glioblastomas on follow-up were significantly higher than those of CNS lymphoma (t-Test, *p* < 0.001), and brain metastasis (t-Test, *p* < 0.01).

The volumes that could be measured in MR perfusion compared with the correlating rapid wash-out volumes revealed no significant differences (*p* > 0.5 for each t-Test “perfusion versus wash-out”) for five subgroups (initial diagnosis: GBM, CNS lymphoma, brain metastasis; and post-therapeutic: GBM and brain metastasis). The only exception were the post-therapeutic CNS lymphomas (*p* < 0.03) where no volumes could be measured in MR perfusion due to the minimal size of the lesions. The additionally calculated analysis of variance of the six subgroups with Tukey’s Test revealed no significant models / results, mainly caused by the low number of cases.

### Intraclass correlation

Intraclass correlation was excellent with ICC (2, k) = 0.979, confidence interval [0.95, 0.99] for all measured volumes (wash-in, wash-out and MR perfusion).

## Discussion

In our feasibility study of 32 patients, we were able to demonstrate the regular, standardized and meaningful use of our new rapid wash-out maps. Even with a short delay of 15 min, we were able to generate useful maps on which the wash-out and wash-in could be easily understood. Semi-automatically volumetrization could be performed easily. We were able to show similarities with MR perfusion, but also revealed differences and potential for additional information.

The new rapid wash-out maps can be volumetrically assessed, are calculated in nearly real-time (10 min), and are optimized for further post-processing, e.g. using AI-tools or neuronal nets.

Especially in cases of radiation necrosis, pseudo-progression and tumor progress, we already found individual cases demonstrating diagnostic differences between wash-out maps and MR perfusion (see Fig. 6).

The new method is also well usable to CNS lymphomas and metastases, although the results, especially in metastases, seem to depend on the histology and need to be investigated in larger studies.

The evaluation of the volumetry did not provide any statistically significant information, since the areas of hyperperfusion and wash-out often differed partially in terms of their location, but differed only slightly in terms of their overall size.

Following RANO (Response Assessment in Neuro-Oncology) criteria [19], and RANO 2.0 [20], for high-grade glioma, in patients with high likelihood of pseudoprogression, mandatory confirmation of progression with a repeat MRI is recommended, volumetric measurements also are an option. This currently applies to therapies with the STUPP scheme [21]. Advance imaging methods, such as ADC (apparent diffusion coefficient)-maps and MR perfusion, are generally recommended. Contrast clearance or late-enhancement is not specifically addressed in these guidelines. RANO for brain metastasis [22] and iRANO (immunotherapy Response Assessment in Neuro-Oncology) [23] for immunotherapy (malignant melanoma, lung cancer, glioblastoma) refine the diagnostic process.

Radiation necrosis and pseudo-progression occur in 10 to 35% of all cases, depending on the study, and represent the main problem in diagnosis [24].

MR perfusion is the most used method to differ progressive disease from radiation necrosis and pseudo-progression [2]. However, there is currently no reliable method of distinguishing between the two [25].

In glioblastoma, the measurement of MR perfusion parameters showed promising results, but seem to vary from study to study, e.g. a study from UK found a high rCBV-threshold of 3.0 [26]. A meta-analysis revealed the following pooled sensitivities and specificities for the differentiation of glioma recurrence and pseudo progression in MR perfusion techniques: 0.82, 0.87 for DSC, 0.83, 0.83 for DCE (Dynamic Contrast Enhanced), and 0.78, 0.86 for ASL (Arterial Spin Labeling) perfusion [27]. Sensitivity and specificity of TRAMs are approximately 0.93 and 0.78 [28].

A combination of ADC and DSC perfusion showed no significant improvement of the diagnostic accuracy compared to each method alone [29]. Post-operative DWI (diffusion-weighted imaging) can be improved by other diffusion weighted imaging methods like STEAM (Stimulated Echo Acquisition Mode) DWI [30], which may increase the diagnostic accuracy after surgery and therefore also in the 3-months follow-up.

A comparative study of MR perfusion versus contrast clearance/TRAMs versus (18 F)-dopa PET/CT showed different advantages and disadvantages of the individual methods, in favor of the two MR methods [7]. Another alternative, MR spectroscopy demonstrated excellent results in some studies [31], but is still matter of debate, because it seems to be relatively susceptible to artifacts and is examiner-dependent.

In immunotherapy for glioblastoma, TRAMs may be useful as well [32]. Some studies also demonstrated that TRAMs can be used in treatment assessment of CNS lymphomas [6, 33].

Treatment-assessment in brain metastasis is also possible after stereotactic surgery [34], but with a larger overlap between tumor recurrence and radiation necrosis. This is possibly due to the delay being too short, so that the medium uptake in metastasis had not yet reached the maximum wash-in and then finally showed the same value as slow-uptake necrosis. Other studies after gamma-knife surgery revealed improved results [35] comparable with MR perfusion [36].

### Limitations

This first study is only intended as a feasibility study. The small number of cases does allow only a few statistically significant statements.

Main limitations of the study were the small sample size, and the partially old protocols and MR scanners. Especially the MR DSC perfusion protocols deviate in some cases considerably from the current consensus recommendations [37].

Additionally, on Siemens MR scanner, we used T1 MPRAGE sequences, optimized for grey-white-matter contrast and not for contrast enhancement. This could reduce the conspicuity of small lesions and reduce the contouring estimates compared with T1 VIBE sequences, as described by Danieli et al. [38].

The short time difference between the contrast-enhanced T1-weighted sequences compared to the TRAMs could reduce the wash-out volume.

In particular, the wash-in/late-enhancement behavior of the different metastasis subtypes is not clear (with low wash-out ratios), but this may also open up new diagnostic perspectives.

In addition, the delay before the first T1-weighted sequence is optimized for glioblastomas. In certain circumstances, a different delay is recommended depending on the histology (possibly also depending on the primary tumor in the case of brain metastases). There is still a big research gap that needs to be filled.

### Outlook

The rapid wash-out is intended as a follow-up test and not just to record the current status. The following studies must show to what extent these rapid wash-out maps can provide useful new information for therapy assessment. It is not expected that these are better than TRAMs. However, they are much easier to integrate into everyday clinical practice, and the question is whether there is any inferiority of our rapid wash-out maps versus TRAMs. We do not expect this to be the case in glioblastomas and CNS lymphomas in particular, and in metastases it probably depends on the subtype. Furthermore, we think that the TRAMs could also be improved by optimizing the timing of the first T1-weighted sequence dependent on the histology. The exact advantages and disadvantages of rapid wash-out-maps compared to MR perfusion and contrast clearance analysis must be investigated in further prospective studies with specific questions.

In future analysis the combination of methods, e.g. DSC perfusion and FDG-PET (Fludeoxyglucose-18 Positron Emission Tomography) for high-grade gliomas [39]; or DTI (Diffusion Tensor Imaging) and DSC perfusion [40], will increase diagnostic safety.

Additional information with possible improvement of diagnostic accuracy could also be achieved by new PET tracers [41–43].

Even in difficult diagnostic tasks, such as the differentiation of multiple space-occupying lesions, the wash-out can provide additional information [44]. A meta-analysis revealed, that combined MRI techniques also enable a better diagnostic accuracy for the treatment response in brain metastasis [45]. However, more prospective and larger studies are needed [46].

The targeted use of AI tools [47] or Machine learning models [48] could also contribute to an improvement as well as the use of new MRI techniques, e.g. T1 mapping [49] for the assessment of standardized values.

## Conclusion

We have successfully tested a new rapid wash out with a delayed first contrast-enhanced T1-weighted sequence. Our results reveal that rapid wash-out maps appear to be at least an equally good marker compared to MR perfusion. Especially the combination of tumor volume and wash-out ratio appears to be a promising method for monitoring disease progression.

## Data Availability

The datasets used and/or analyzed during the current study available from the corresponding author on reasonable request.

## Abbreviations

ADC: Apparent Diffusion Coefficient
AI: Artificial Intelligence
ASL: Arterial Spin Labeling (MR perfusion)
BM: Brain Metastases
CCA: Contrast Clearance Analysis
DCE: Dynamic Contrast Enhanced (MR perfusion)
DSC: Dynamic Susceptibility Contrast (MR perfusion)
DTI: Diffusion Tensor Imaging
DWI: Diffusion-Weighted Imaging
FDG-PET: Fludeoxyglucose-18 Positron Emission Tomography
Gb: Glioblastoma
iRANO: immunotherapy Response Assessment in Neuro-Oncology
MPRAGE: Magnetization Prepared with RApid Gradient Echo
MRI: Magnetic Resonance Imaging
MR: Magnetic Resonance
rCBV: relative Cerebral Blood Volume
RANO: Response Assessment in Neuro-Oncology
SD: Standard Deviation
STEAM: Stimulated Echo Acquisition Mode
TRAMs: Treatment Response Assessment Maps
VIBE: Volumetric Interpolated Breath-hold Examination
WHO: World Health Organization
